# Uncovering the pathogenesis of obesity complicated with papillary thyroid carcinoma via bioinformatics and experimental validation

**DOI:** 10.18632/aging.204993

**Published:** 2023-09-05

**Authors:** Kaisheng Yuan, Di Hu, Xiaocong Mo, Ruiqi Zeng, Bing Wu, Zunhao Zhang, Ruixiang Hu, Cunchuan Wang

**Affiliations:** 1 Department of Metabolic and Bariatric Surgery, The First Affiliated Hospital of Jinan University, Guangzhou 510630, Guangdong, China; 2 Guangdong-Hong Kong-Macao Joint University Laboratory of Metabolic and Molecular Medicine, The University of Hong Kong and Jinan University, Guangzhou 510630, Guangdong, China; 3 Department of Neurology and Stroke Centre, The First Affiliated Hospital of Jinan University, Jinan University, Guangzhou 510630, Guangdong, China; 4 Department of Oncology, The First Affiliated Hospital of Jinan University, Jinan University, Guangzhou 510630, Guangdong, China; 5 Department of Urology Surgery, The Second People’s Hospital of Yibin City, Yibin 644002, Sichuan, China; 6 Department of Pathology, The First Affiliated Hospital of Jinan University, Jinan University, Guangzhou 510630, Guangdong, China

**Keywords:** obesity, papillary thyroid carcinoma, bioinformatics, differentially expressed genes, hub genes

## Abstract

This study aimed to investigate the common molecular mechanism between obesity and papillary thyroid cancer (PTC), the most common pathological type of thyroid cancer. In this study, we obtained gene expression datasets for obesity (GSE151839) and PTC (GSE33630) from the Gene Expression Omnibus (GEO). We used the Perl program and R software to identify differentially expressed genes (DEGs) and common genes, perform GO function and KEGG pathway enrichment analysis, construct a protein-protein interaction (PPI) network, identify hub genes, and perform transcription factors (TFs) analysis. After undergoing validation in external datasets and *in vitro* experiments, common targets for both diseases were ultimately identified. A total of 23 genes that were differentially expressed (DEGs) between obesity and papillary thyroid carcinoma (PTC) were identified in our study. Among these DEGs, 17 genes were up-regulated while 6 genes were down-regulated. Then the top ten key genes were identified from the PPI network using cytoHubba and MCODE plug-in. Further evidence from external datasets revealed that MMP9, MNDA, TNC, and CHIT1 were identified as hub genes for both diseases. The study utilized Transcriptional Regulatory Relationships Unraveled by Sentence-based Text mining (TRRUST) to perform an enrichment analysis of TFs. This analysis identified ELF4 and STAT3 as common TFs for both diseases. Additionally, *in vitro* experiments were conducted to further analyze the clinical significance and biological functions of these TFs. The identification and investigation of hub genes and their corresponding TFs that regulate abnormalities in obesity and PTC can enhance our comprehension of the underlying connection between these two diseases, thus leading to the development of novel diagnostic approaches.

## INTRODUCTION

According to the World Health Organization (WHO), obesity is characterized by an abnormal or excessive accumulation of fat that can cause health problems. It is considered a chronic metabolic disease caused by a combination of factors and is often linked to weight gain and metabolic abnormalities. The commonly used international standard for measuring obesity is the body mass index (BMI). A BMI of 25.0-29.9 kg/m^2^ is considered overweight, while a BMI of ≥30.0 kg/m^2^ is considered obese [1]. According to research, one-third of the global population is either overweight or obese, resulting in 3.4 million deaths annually due to obesity-related complications [2]. Furthermore, studies have shown that obesity is a significant risk factor for various types of cancer, such as colorectal, postmenopausal breast, endometrial, thyroid, esophageal, pancreatic, and liver cancer [3]. In a meta-analysis of 21 studies comprising 12,199 cases of thyroid cancer, overweight individuals had a 25% increased risk of thyroid cancer while obese individuals had a 55% increased risk compared to individuals with normal weight [4].

Thyroid cancer (TC) is a rapidly growing malignancy [5], representing 3.8% of all diagnosed tumors annually [6]. The most prevalent pathological type is papillary thyroid cancer (PTC), accounting for 86% of all thyroid cancers [7]. Although obesity has been identified as a high-risk factor for PTC, the molecular mechanisms linking obesity and PTC remain unclear [8]. The MAPK pathway is believed to be the main pathway in the development of thyroid cancer, specifically PTC. This pathway is responsible for a variety of secondary molecular alterations that work together to enhance the oncogenic activity of the pathway, ultimately leading to the upregulation of several oncogenic proteins [9]. Obesity is characterized by chronic inflammation, which can disrupt the normal functioning of mitochondria and lead to an overproduction of reactive oxygen species (ROS). These ROS can then activate the MAPK pathway, ultimately promoting the development and invasion of PTC [10, 11].

This study aims to accurately understand the relationship between obesity and PTC by identifying their potential molecular regulatory mechanisms and targets through a combination of bioinformatics and experimental validation. The datasets of obesity and PTC were downloaded from the Gene Expression Omnibus (GEO), and the differentially expressed genes (DEGs) of both were screened to obtain the common DEGs of both. These results offer novel insights into the shared pathogenesis of obesity and PTC. To identify the targets linking obesity and PTC, we conducted GO and KEGG enrichment analysis, protein-protein interaction (PPI) network analysis, and transcription factors (TFs) enrichment analysis. Experimental validation was then performed to confirm the identified targets. Our study ultimately yielded a list of key genes and TFs that can aid in predicting and diagnosing patients with both diseases.

## RESULTS

### Identification of DEGs

**Figure 1:**
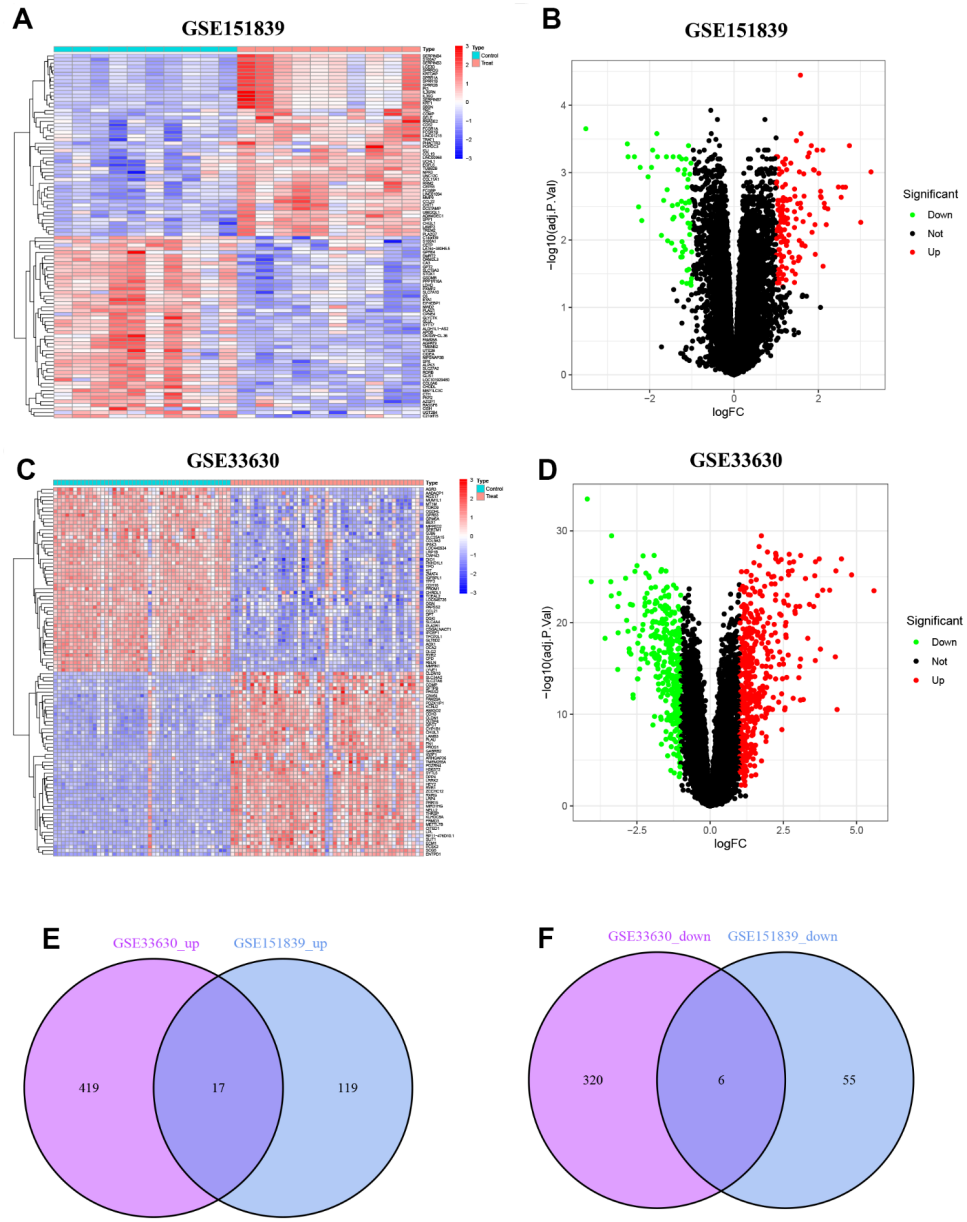
**Identification of DEGs.** (**A**) Heatmap of DEGs in GSE151839. (**B**) Volcano plot of DEGs in GSE151839. (**C**) Heatmap of DEGs in GSE33630. (**D**) Volcano plot of DEGs in GSE33630. (**E**) Venn diagram shown the 17 up-regulated DEGs between GSE151839 and GSE33630 datasets. (**F**) Venn diagram shown the 6 down-regulated DEGs between GSE151839 and GSE33630 datasets.

In GSE151839, a total of 197 DEGs were identified, consisting of 136 up-regulated genes and 61 down-regulated genes. These DEGs were visualized in both the heatmap and volcano map (Figure 1A, 1B). Likewise, in GSE33630, a total of 762 DEGs were identified, with 436 up-regulated genes and 326 down-regulated genes, which were also visualized in both the heatmap and volcano map (Figure 1C, 1D). By taking the intersection of the two groups of DEGs, a total of 23 common DEGs were obtained, with 17 being up-regulated (Figure 1E) and 6 being down-regulated (Figure 1F).

### GO and KEGG functional enrichment analysis for DEGs

**Figure 2:**
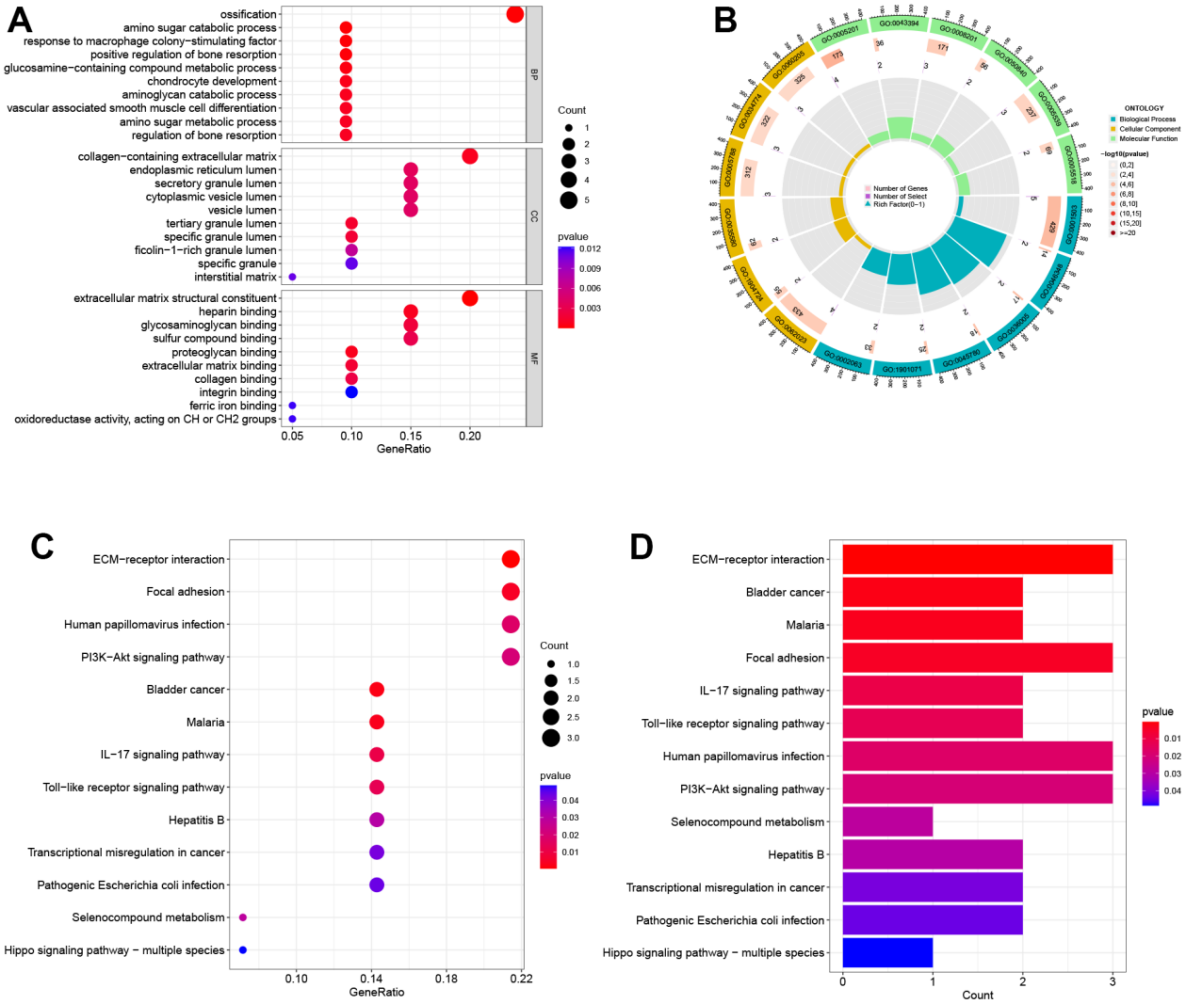
**Biological functional enrichment research of DEGs.** (**A**) GO enrichment analysis of DEGs. (**B**) GO circle plots showed the enrichment results of the top 20 GOs. (**C**, **D**) KEGG enrichment analysis of DEGs.

The results of GO analysis show that DEGs are primarily involved in three categories: biological process (BP), cellular component (CC), and molecular function (MF). Specifically, the BP category indicates that the DEGs are mainly involved in ossification, amino sugar catabolic process, and response to macrophage colony-stimulating factor. In the MF category, the DEGs are mainly associated with cell membrane-related functions, such as collagen-containing extracellular matrix, endoplasmic reticulum lumen, and secretory granule lumen. According to the CC analysis, the enrichment of DEGs was significant in extracellular matrix structural constituent, heparin binding, and glycosaminoglycan binding, as shown in Figure 2A. The top 20 GO enrichment results were further visualized using GO circle plots in Figure 2B. The KEGG pathway analysis revealed that these genes were mainly involved in ECM-receptor interaction, focal adhesion, and human papillomavirus infection pathways. The findings indicate that these pathways could be pivotal in the development and progression of obesity and PTC (Figure 2C, 2D).

### The construction of PPI network, module analysis and identification of hub genes

**Figure 3:**
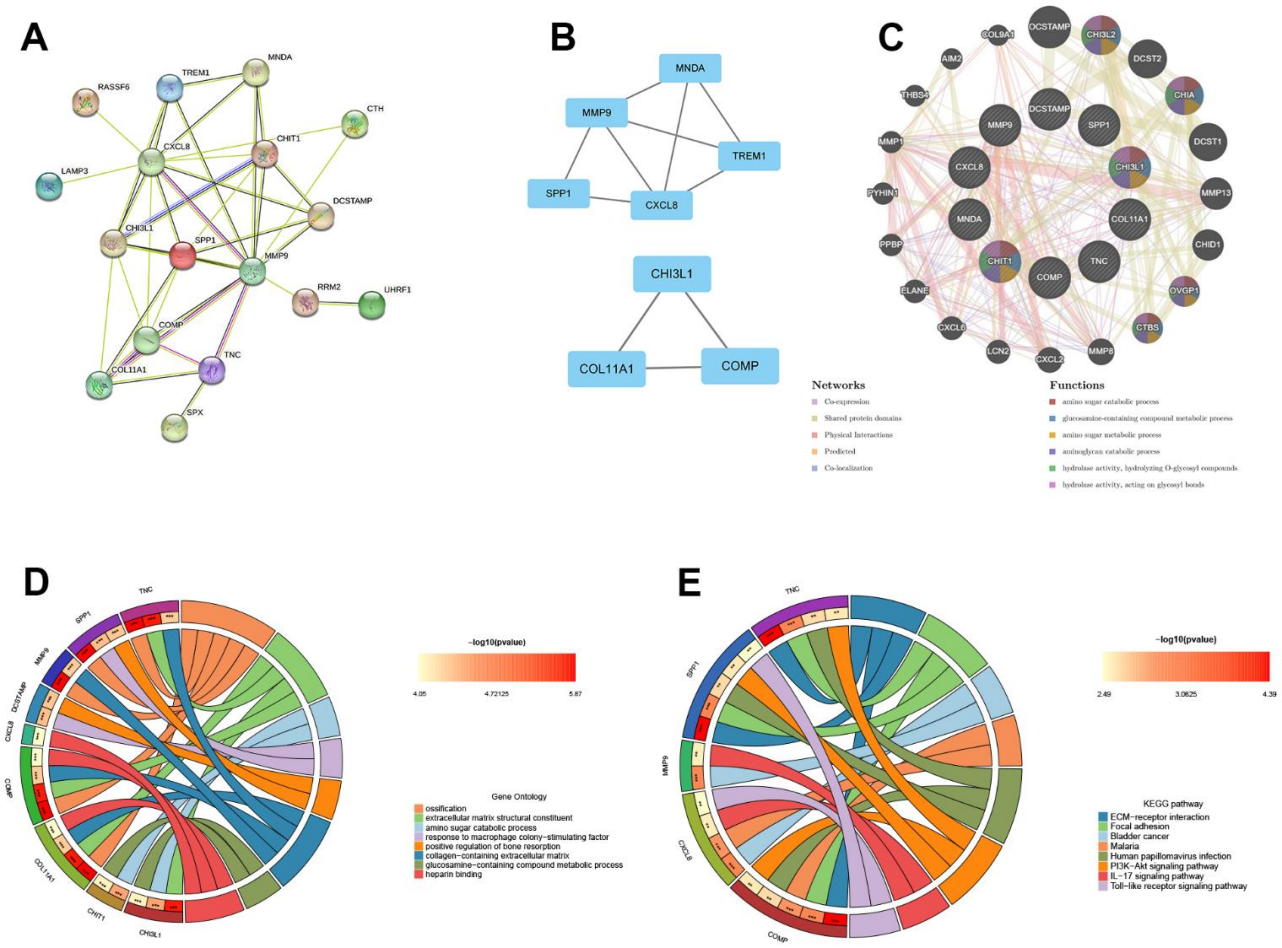
**The construction of PPI network, module analysis and identification of hub genes.** (**A**) The PPI network of common DEGs. (**B**) Two tightly connected modules. (**C**) The 10 hub genes and 20 interacting genes were functionally analyzed by the GeneMania database. (**D**) GO enrichment analysis of hub genes. (**E**) KEGG enrichment analysis of hub genes.

To analyze the PPI networks of common DEGs, we utilized STRING and Cytoscape, as depicted in Figure 3A. The PPI network was composed of 22 nodes and 39 edges. We further used the MCODE plug-in in Cytoscape to decompose the PPI network and obtain 2 tightly connected modules, as shown in Figure 3B. Additionally, we employed the cytoHubba plug-in of Cytoscape to identify the top 10 hub genes based on degree-ranking, which were MMP9, CXCL8, SPP1, CHI3L1, CHIT1, COMP, COL11A1, TNC, MNDA, and DCSTAMP.

In this study, the 10 hub genes and 20 interacting genes were subjected to functional analysis and a co-expression network was constructed using the GeneMania database. The 10 hub genes are represented in the inner circle, while the outer circle represents the genes that are connected to the hub genes. Figure 3C demonstrates that these genes are primarily enriched in amino sugar catabolic process, glucosamine-containing compound metabolic process, amino sugar metabolic process, aminoglycan catabolic process, hydrolase activity, hydrolyzing O-glycosyl compounds, hydrolase activity (hydrolyzing O-glycosyl compounds), and hydrolase activity (acting on glycosyl bonds). The GO enrichment analysis results showed that hub genes were predominantly enriched in ossification, extracellular matrix structural constituent, amino sugar catabolic process, among other processes (Figure 3D). This was further supported by the results of KEGG enrichment analysis, which also showed significant enrichment of these genes in pathways related to ECM-receptor interaction, focal adhesion, bladder cancer, etc. (Figure 3E).

### Validation of hub genes expression in external datasets

**Figure 4:**
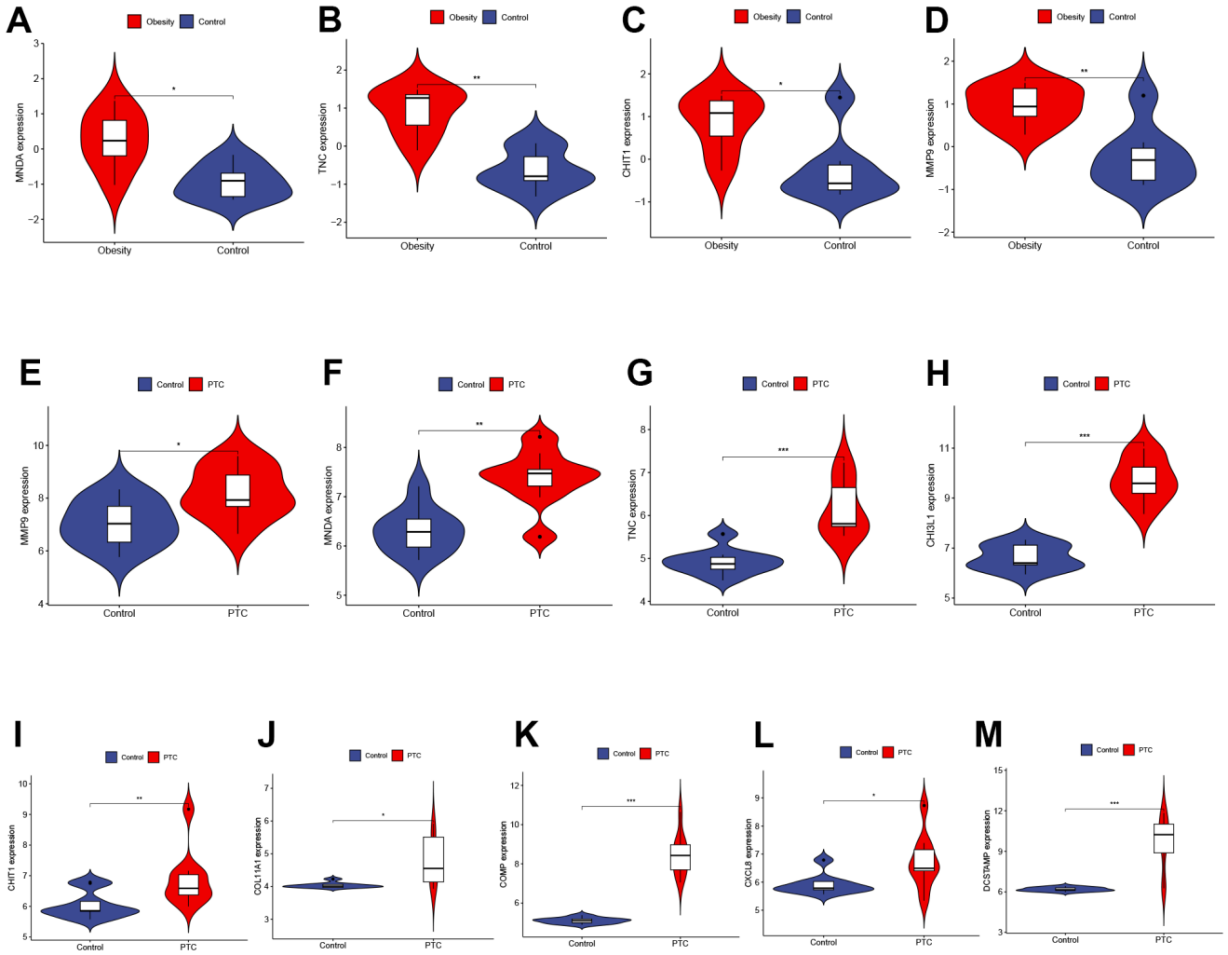
**Validation of hub genes expression in external datasets.** (**A**–**D**) The expression of MNDA, TNC, CHIT1, and MMP9 in GSE44000 dataset ((**A**) P < 0.05, (**B**) P < 0.01, (**C**) P < 0.05, (**D**) P < 0.01). (**E**–**M**) The expression of MMP9, MNDA, TNC, CHI3L1, CHIT1, COL11A1, COMP, CXCL8, and DCSTAMP in GSE3467 dataset ((**E**) P < 0.05, (**F**) P < 0.01, (**G**) P < 0.001, (**H**) P < 0.001, (**I**) P < 0.01, (**J**) P < 0.05, (**K**) P < 0.001, (**L**) P < 0.05, (**M**) P < 0.001). P < 0.001 was denoted as “***”, P < 0.01 as “**”, P < 0.05 as “*”, and P > 0.05 as **“**ns**”**.

To validate the reliability of Hub Genes, we selected additional datasets related to obesity and PTC. In the GSE44000 dataset, we observed a significant upregulation of MNDA, TNC, CHIT1, and MMP9 in obese tissues (Figure 4A–4D). In the GSE3467 dataset, we found that the expression of MMP9, MNDA, TNC, CHI3L1, CHIT1, COL11A1, COMP, CXCL8, and DCSTAMP was significantly upregulated in PTC as compared to normal tissues (Figure 4E–4M). In conclusion, these four genes (MNDA, TNC, CHIT1, MMP9) may serve as a link between obesity and PTC and should be further examined in subsequent analyses. P < 0.001 was denoted as “***”, P < 0.01 as “**”, P < 0.05 as “*”, and P > 0.05 as “ns”.

### Identification and validation of TFs

**Figure 5:**
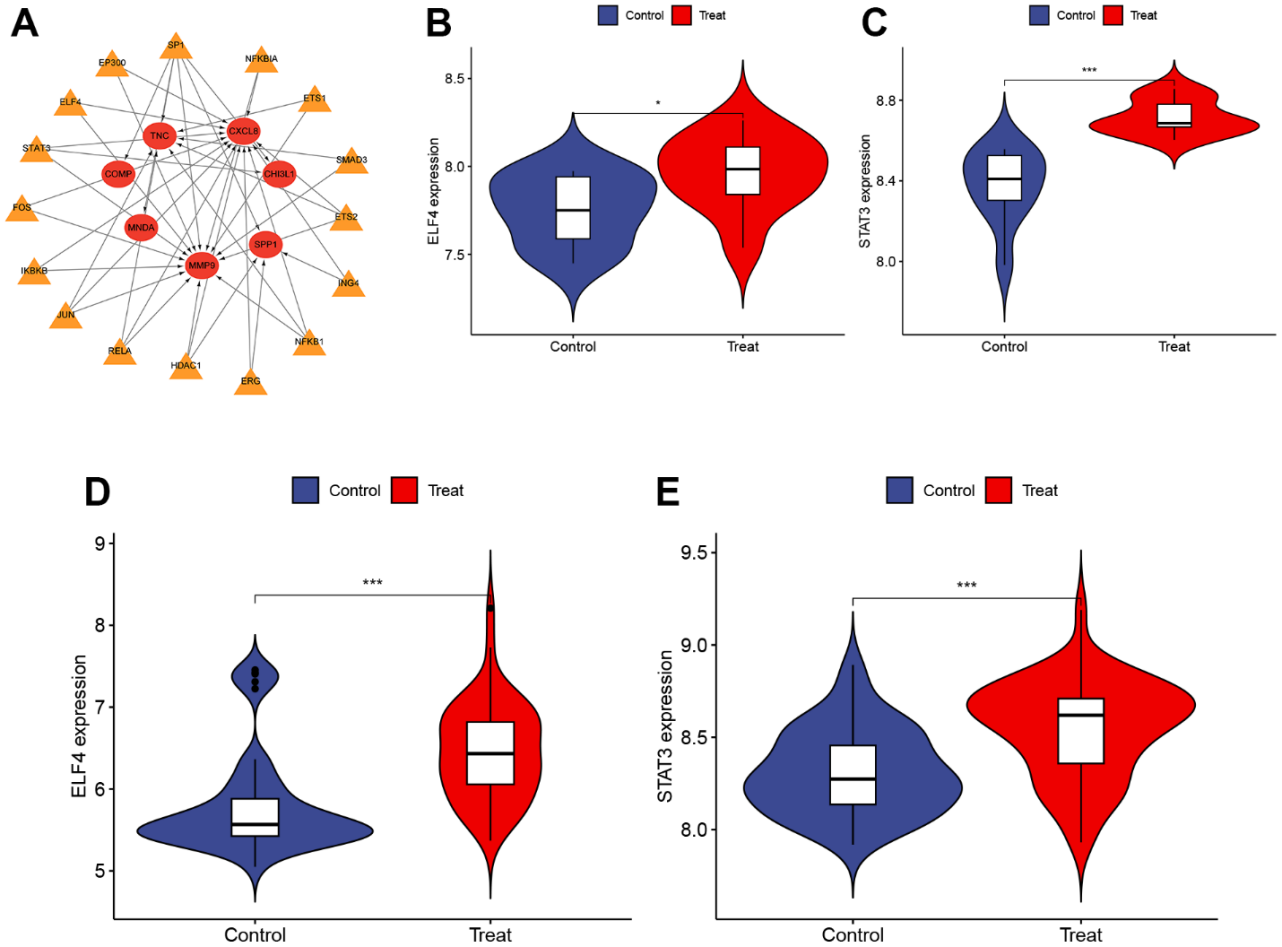
**Identification and validation of TFs.** (**A**) The TFs regulatory network. Red represents the hub genes and yellow represents the TFs. (**B**, **C**) The expression of ELF4 and STAT3 in GSE151839 dataset ((**B**) P < 0.05, (**C**) P < 0.001). (**D**, **E**) The expression of ELF4 and STAT3 in GSE33630 dataset ((**D**) P < 0.001, (**E**) P < 0.001). P < 0.001 was denoted as “***”, P < 0.01 as “**”, P < 0.05 as “*”, and P > 0.05 as **“**ns**”**.

This study utilized the TRRUST database to predict the TFs in DEGs. A total of 16 TFs were identified to play a role in DEGs (Figure 5A). These TFs were further validated in both obesity (GSE151839) and PTC (GSE33630) datasets. Figure 5B–5E depicted that both datasets had high expression levels of ELF4 and STAT3, which were responsible for regulating three hub genes (MMP9, CXCL8, CHI3L1). P < 0.001 was denoted as “***”, P < 0.01 as “**”, P < 0.05 as “*”, and P > 0.05 as “ns”.

### Experimental validation

**Figure 6:**
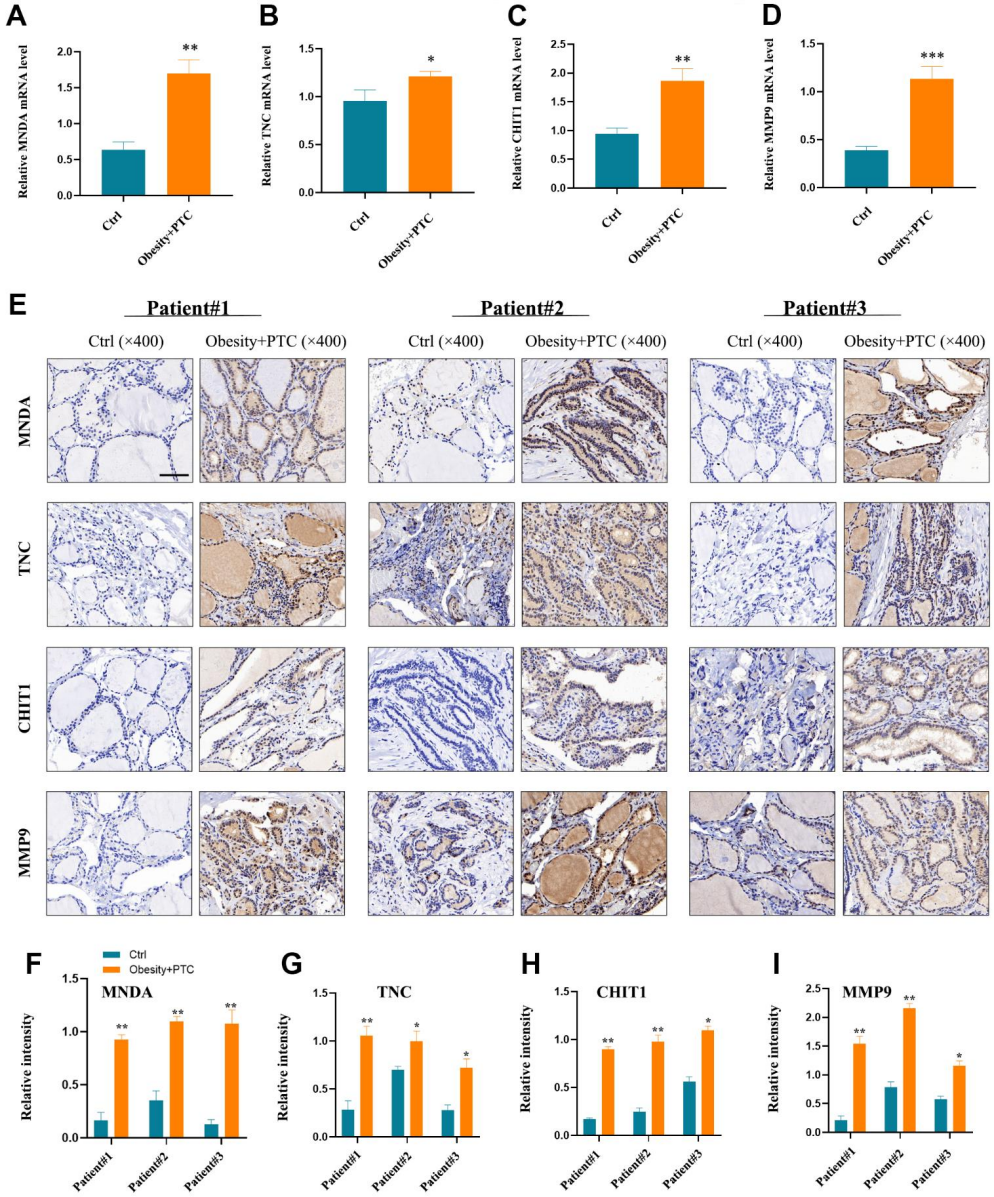
**Experimental validation of hub genes.** (**A**–**D**) MNDA, TNC, CHIT1 and MMP9 were found to be highly expressed in patients’ serum with obesity combined with PTC ((**A**) P < 0.01, (**B**) P < 0.05, (**C**) P < 0.01, (**D**) P < 0.001). (**E**–**I**) The expression of four hub genes in the patient tissues and the normal tissues (**F**) patient1: P < 0.01, patient2: P < 0.01, patient3: P < 0.01; (**G**) patient1: P < 0.01, patient2: P < 0.05, patient3: P < 0.05; (**H**) patient1: P < 0.01, patient2: P < 0.01, patient3: P < 0.05; (**I**) patient1: P < 0.01, patient2: P < 0.01, patient3: P < 0.05). P < 0.001 was denoted as “***”, P < 0.01 as “**”, P < 0.05 as “*”, and P > 0.05 as **“**ns**”**.

The analysis of the patient’s serum revealed high expression levels of MNDA, TNC, CHIT1, and MMP9 in patients with obesity combined with PTC, as shown in Figure 6A–6D. Moreover, the expression of these genes was found to be higher in tumor tissues compared to normal tissues, as depicted in Figure 6E–6I. P < 0.001 was denoted as “***”, P < 0.01 as “**”, P < 0.05 as “*”, and P > 0.05 as “ns”.

## DISCUSSION

The prevalence of obesity is increasing globally due to improvements in living standards and changes in lifestyle. According to the World Health Organization (WHO), in 2014 there were over 1.9 billion overweight individuals worldwide, with more than 600 million being classified as obese. Among adults aged 18 and above, the proportion of overweight and obese individuals was 39% and 13%, respectively [12]. Over the past decade, TC has become one of the most prevalent malignancies of the endocrine system, with its incidence increasing annually [13]. Accounting for about 70% of TC cases, PTC is the most common histological type [14]. Recently, obesity and TC have become hot topics in the field of endocrinology. Adequate evidence has shown that obesity is a risk factor for thirteen malignant tumors, including thyroid cancer [15]. Research has shown that as BMI increases, the malignancy of tumors in PTC patients also increases. This is evident pathologically through an increased risk of tumor invasion and is strongly correlated with both tumor size and stage. Patients with higher BMI tend to have larger tumor volumes and are more likely to be diagnosed with stage III or IV PTC [16, 17].

Based on current clinical and basic research, there are several potential mechanisms through which obesity may promote the onset and progression of PTC. These mechanisms involve hyperinsulinemia, heightened aromatase activity, abnormal secretion of obesity factors, chronic inflammatory response, immune response, and oxidative stress [18, 19]. This study utilized bioinformatics and experimental validation to identify the common DEGs and TFs between obesity and PTC for the first time. This discovery has significant implications as it can aid in the development of new biomarkers and effective therapeutic targets for both diseases, ultimately improving patient prognosis.

This study utilized bioinformatics to identify hub genes associated with both obesity and PTC, and conducted a comprehensive analysis to elucidate the potential molecular mechanisms linking the two conditions. The analysis of the GSE151839 and GSE33630 datasets revealed 23 common DEGs, comprising of 17 up-regulated and 6 down-regulated genes. The subsequent GO and KEGG enrichment analysis indicated that these genes were predominantly enriched in inflammation-related pathways. To construct the PPI network, we selected 10 central genes (MMP9, CXCL8, SPP1, CHI3L1, CHIT1, COMP, COL11A1, TNC, MNDA, DCSTAMP) that have a close relationship to both obesity and PTC. We then verified their diagnostic efficiency in external datasets (P < 0.05). Our results indicate that in the GSE44000 obesity-related dataset, the expression levels of MNDA, TNC, CHIT1, and MMP9 exhibited significant differences. The GSE3467 PTC-related dataset revealed significant differences in the expression of MMP9, MNDA, TNC, CHI3L1, CHIT1, COL11A1, COMP, CXCL8, and DCSTAMP. Based on our findings, it can be concluded that these four genes (MNDA, TNC, CHIT1, and MMP9) may have important biological roles in preventing and treating obesity and PTC. Furthermore, our analysis of the TRRUST database identified 16 TFs that play a role in the differentially expressed genes. Further validation revealed that in obesity and PTC, only ELF4 and STAT3 showed high expression levels. These genes were found to regulate three hub genes, namely MMP9, CXCL8, and CHI3L1. Among these hub genes, MMP9 was the only one that was differentially expressed and regulated by TFs in both obesity and PTC datasets.

MMP9 is a member of the matrix metalloprotein (MMP) family and is located on chromosome 20q11.1~13.1, spanning 26~27kbp with 13 exons and 9 introns [20]. It plays a crucial role in various physiological processes including embryonic development, reproduction, vascular formation, skeletal development, wound healing, cell migration, learning, and memory. Additionally, it is involved in pathological processes associated with extracellular matrix degradation [21]. Multiple studies have demonstrated that MMP9 is notably increased in malignant tumors, including colon cancer, gastric cancer, lung cancer, breast cancer, and cervical cancer. As a result, it has become a potential target for anti-tumor drugs [22–26]. In a study conducted by Maryam et al. in Iran, 60 patients with PTC and 30 patients with benign multinodular goiter (MNG) were compared, and it was found that the levels of MMP9 protein in tumor tissues were significantly higher than in adjacent non-tumor tissues (P < 0.001). Compared to patients with benign multinodular goiter, those with papillary thyroid cancer (PTC) showed a significant increase (P = 0.004) in MMP9 levels [27]. Additionally, research by Marecko et al. suggests that MMP9 not only serves as a biomarker for PTC, but also plays a role in promoting the migration and invasion of thyroid cancer cells [28]. Taken together, these studies suggest that MMP9 is a biomarker for TC and may contribute to its high aggressiveness and poor prognosis. Schaschkow et al. reported an enhanced cytoplasmic expression of STAT3 in severely obese individuals with diabetes. Furthermore, a meta-analysis incorporating eight studies, encompassing 448 thyroid cancer patients and 227 controls, revealed a significant correlation between STAT3 protein expression and susceptibility to thyroid cancer, as well as clinical-pathological characteristics. These findings suggest that STAT3 may serve as a potential predictive factor for the clinical progression of thyroid cancer [29, 30]. To the best of our knowledge, the literature has not reported on the role of ELF4 in thyroid cancer and obese patients. Investigating the involvement of ELF4 in these contexts represents a future research direction for our study.

In this study, we investigated the expression patterns and regulatory mechanisms of a specific diagnostic gene. While our research has provided valuable insights into the gene’s characteristics, it is important to acknowledge certain limitations that should be considered. One major limitation of our study is the absence of experimental validation for the predicted TFs corresponding to the gene. Although we described these factors based on computational analysis and existing literature, their direct binding to the gene was not experimentally verified. Consequently, alternative interpretations and variations in their regulatory interactions remain possible. Future research should prioritize experimental validation of the predicted TFs. This experimental validation will provide robust evidence and enhance our understanding of the gene’s regulatory network.

This study presents the first exploration of the common hub genes and TFs of obesity and PTC using bioinformatics and experimental validation. The expression of four common hub genes (MNDA, TNC, CHIT1, and MMP9) in both patients’ serum and tissues was validated *in vitro*. Further research is needed to investigate the mechanisms between these hub genes and TFs.

## CONCLUSIONS

In this study, we constructed a co-expression network between obesity and PTC and identified four common hub genes (MNDA, TNC, CHIT1, MMP9) and two common TFs (ELF4, STAT3), which might provide new diagnostic and therapeutic strategies for obesity-related PTC.

## MATERIALS AND METHODS

**Figure 7:**
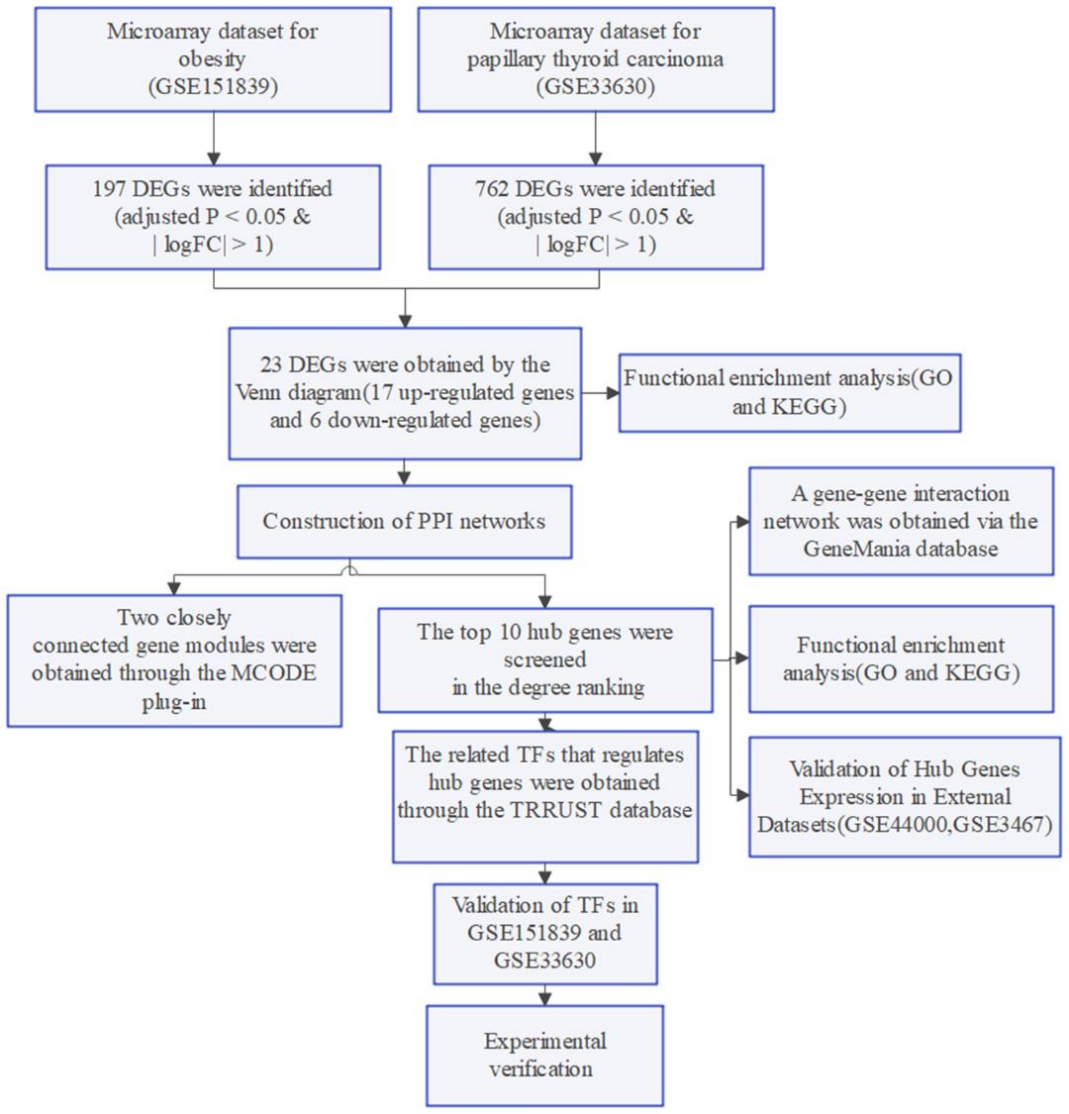
Flow chart of the study.

The flow chart of this study is shown in Figure 7.

### Dataset collection and process

Gene expression profiles were searched in the GEO database using the keywords “obesity” and “papillary thyroid carcinoma”, respectively. Inclusion criteria for the datasets: (1) gene expression analysis must include both case and control groups; (2) the tissue used for sequencing is whole blood from humans; (3) raw or processed data must be available for re-analysis. Four datasets (GSE151839, GSE44000, GSE33630, GSE3467) were ultimately downloaded. GSE151839 (obesity: 10 cases; control: 10 cases) was executed on the GPL570 platform and GSE44000 (obesity: 7 cases; control: 7 cases) was executed on the GPL6480 platform. GSE33630 (PTC: 49 cases; control: 45 cases) and GSE3467 (PTC: 9 cases; control: 9 cases) were both executed on the GPL570 platform. GSE151839 and GSE33630 were used as the training group, while the external validation group consisted of GSE44000 and GSE3467.

For gene expression profiling, the series matrix files of the dataset underwent log2 transformation. Then, the probes were matched to their respective gene symbols using the annotation files of the corresponding platforms. This resulted in gene matrices with column names for genes and row names for samples, which were used for subsequent analysis.

### Identification of DEGs

DEGs between the case and control groups for obesity and PTC were obtained using the “limma” package in R software (version 4.2.2). Cutoff conditions for adjusted P < 0.05 and |logFC| > 1 were set. DEGs were identified separately for each dataset, and the common DEGs were identified using a Venn diagram. (https://goodcalculators.com/venn-diagram-maker/).

### Functional enrichment analysis of DEGs

To analyze the shared differentially expressed genes, we used the R package ‘Clusterprofiler’ to perform GO functional enrichment analysis and KEGG pathway enrichment analysis. For the GO functional enrichment analysis, we divided the analysis into three parts: gene ontology process (BP), cellular component (CC), and molecular function (MF). We visualized the GO term using the ‘GOplot’ package, and adjusted P value < 0.05 was considered significant.

### The construction of PPI network and identification of hub genes

To observe the common functional characteristics of hub genes for DEGs, we established a PPI network using STRING (https://cn.string-db.org/), a search tool for interacting genes. The minimum required interaction score was set at a low confidence level of 0.150. The resulting network was visualized using Cytoscape 3.9.1 (https://cytoscape.org). We utilized the MCODE plug-in within Cytoscape to filter and identify functional modules within the PPI network. Our degree cutoff was set to 2, node score cutoff to 0.2, k-core to 2, and max depth to 100. The topological parameters of each node within the PPI network were then calculated using the cytoHubba plug-in, and we filtered the top 10 hub genes based on degree. To construct a gene-gene interaction network of 10 hub genes and their neighboring genes, we utilized the GeneMania online database (http://www.genemania.org). Subsequently, we performed GO functional enrichment analysis and KEGG pathway enrichment analysis for the 10 hub genes by employing the “Clusterprofiler” package.

### Validation of hub genes expression in external datasets

To investigate the potential use of the ten hub genes as biomarkers for patients with obesity and PTC, we will verify their mRNA expression in two datasets, GSE44000 (obesity: 7 cases; control: 7cases) and GSE3467 (PTC: 9 cases; control:9 cases). The mRNA expression data will be visualized through box plots, which will be created using the ‘ggpubr’ package in R software.

### Identification and validation of TFs

The transcriptional regulatory relationships unraveled by sentence-based text mining (TRRUST) database version 2.0 (https://www.grnpedia.org/trrust/) is a human and mouse transcriptional regulatory network that contains information on TFs and their regulated hub genes. The TRRUST database was utilized in our study to analyze TFs linked to hub genes. Subsequently, we created a gene-TFs network through Cytoscape. To verify the expression of TFs in GSE151839 and GSE33630 datasets, we utilized the ‘ggpubr’ package in R software.

### Patients and samples

25 pairs of matched PTC and adjacent normal tissues were collected during the initial operation of obese patients with thyroid cancer in this study (Supplementary Table 1). Prior to surgery, 5 ml of blood samples were collected from all fasting subjects and placed directly into tubes containing sodium citrate. The blood samples were then centrifuged at 4,000 × g for 10 minutes at 4° C. All serum and tissue samples were snap-frozen in liquid nitrogen and stored at -80° C until extraction. All tissue specimens were confirmed through postoperative histopathological examination. The Ethics Committee of the First Affiliated Hospital of Jinan University approved all procedures involving human subjects in this study. Additionally, all patients provided informed consent prior to participation.

### RT−qPCR

In this study, we extracted total RNA from the serum of patients with obesity combined with PTC. Subsequently, the RNA was reverse-transcribed into complementary DNA (cDNA) employing the SuperScript VILO cDNA Kit from Thermo Fisher Scientific, Inc. We analyzed the results using the 2-ΔΔCt technique and the primers used are listed in Table 1.

**Table 1:** Primer list.

**Gene**	**Primers**
MNDA	Forward: 5’- ACTGACATCGGAAGCAAGAGGG -3’ Reverse: 5’- TGCAGATGTGCTGGCTCCTGAG -3’
TNC	Forward: 5’- ATGTCCTCCTGACAGCCGAGAA -3’ Reverse: 5’- AGTCACGGTGAGGTTTTCCAGC -3’
CHIT1	Forward: 5’- AGCACCACTGAGTGGAATGACG -3’ Reverse: 5’- TGAGTGCCGAAATTCCAGCCTC -3’
MMP9	Forward: 5’- GCCACTACTGTGCCTTTGAGTC -3’ Reverse: 5’- CCCTCAGAGAATCGCCAGTACT -3’
GAPDH	Forward: 5’- GTCTCCTCTGACTTCAACAGCG -3’ Reverse: 5’- ACCACCCTGTTGCTGTAGCCAA -3’

### Immunohistochemistry (IHC)

The tissues were initially stored in 4% paraformaldehyde for 15 minutes, then soaked in paraffin and cut into 4 μm sections. Antigens were extracted after the process of dewaxing and dehydration. Next, the sections were fixed with 3% hydrogen peroxide and blocked with 5% bovine serum albumin (BSA) for 15 minutes at room temperature. The anti-MNDA (ab188566; 1:300; Abcam), anti-TNC (#93029; 1:500; CST), anti-CHIT1 (PA5-109528; 1:100; Invitrogen) and anti-MMP9 (#13667; 1:400; CST) were then incubated overnight at 4° C. Finally, after 3 to 15 minutes of color development with chromogen, the sections were photographed under a light microscope.

### Statistical analysis

Bioinformatics analysis was conducted using the Perl (version 5.30.0) program and R software (version 4.2.2). SPSS 25.0 software and GraphPad Prism 8.0.1 were utilized for statistical analyses. Differences were assessed using One-way analysis of variance (ANOVA) and Student’s t-test. Each experiment was independently repeated three times. A significance level of P < 0.05 was used for all statistical analyses.

### Availability of data and materials

Data will be made available on request.

### Consent for publication

 All the authors are aware and given consent on the submission and publication of this article.

## Supplementary Material

Supplementary Table 1
