# Supplementary material for: Uncovering the pathogenesis of obesity complicated with papillary thyroid carcinoma via bioinformatics and experimental validation
Source: Aging (Albany NY). 2023 Sep 5;15(17):8729–43. doi: 10.18632/aging.204993 (PMC10522395; doi:10.18632/aging.204993)
Supplement: Supplementary Table 1 [file aging-15-204993-s001.pdf]

SUPPLEMENTARY TABLE

Supplementary Table 1. Patients' characteristics.

Patient ID	Age (year)	Sex	Height (cm)	Weight (kg)	BMI (kg/m^2)	Pathological stage	Clinical stage
1	23	Female	158.3	88.3	'35.2369957328517	T1bN1M0	I
2	29	Female	160.3	93.6	'36.4257752833213	T1aN1M0	I
3	32	Male	180.3	105.3	'32.3919368993995	T2N1M1	II
4	57	Female	165.7	97.3	'35.4378873628785	T4aN1M0	III
5	25	Male	171.4	144.8	'49.2886504032275	T1bN1M0	I
6	58	Male	180.7	137.4	'42.0794861280105	T3N1M0	II
7	59	Male	174.2	134.8	'44.4215379870057	T4aN1M0	III
8	50	Female	150.7	86.7	'38.1761908263538	T3aN1M1	II
9	62	Female	155.6	108.2	'44.6897654654674	T4bN1M0	IVA
10	34	Male	171.2	104.6	'35.6881605380383	T2N1M0	I
11	66	Female	166.1	90.2	'32.6939408558636	T4aN1M0	III
12	37	Female	162.3	123.4	'46.8466047031106	T1bN1M1	II
13	26	Male	170.8	101.8	'34.8957105013465	T2N1M1	II
14	63	Female	158.7	91.1	'36.1713338010593	T4bN1M1	IVB
15	56	Female	158.1	82	'32.8057606915774	T4aN1M0	III
16	27	Male	180.6	137.1	'42.0341203003646	T1aN1M0	I
17	27	Female	157.4	93.4	'37.699658846342	T1aN0M0	I
18	55	Male	184.5	130.5	'38.336968735541	T4bN1M0	IVA
19	44	Female	168	84.2	'29.8327664399093	T4aN1M1	II
20	19	Male	166.2	91	'32.9442294018921	T1aN0M0	I
21	27	Female	162.8	111.6	'42.1071059891699	T3bN1M1	II
22	34	Female	156.6	82.4	'33.6003418753232	T1aN0M0	I
23	35	Female	163.6	101.2	'37.8106300177546	T3aN1M1	II
24	21	Male	174.7	111.5	'36.5333129751583	T1aN0M0	I
25	23	Female	166.2	94.1	'34.0665053485499	T2N1M0	I
